# Comparative lipid profiling dataset of the inflammation-induced optic nerve regeneration

**DOI:** 10.1016/j.dib.2019.103950

**Published:** 2019-04-25

**Authors:** Anna Trzeciecka, David T. Stark, Jacky M.K. Kwong, Maria Piqueras, Sanjoy K. Bhattacharya, Joseph Caprioli

**Affiliations:** a Bascom Palmer Eye Institute, Miller School of Medicine at University of Miami, Miami, FL 33136, USA; b Stein Eye Institute, David Geffen School of Medicine at UCLA, Los Angeles, CA 90095, USA

**Keywords:** Optic nerve, Retina, Optic nerve crush, Regeneration, Zymosan, Lipid profile

## Abstract

In adult mammals, retinal ganglion cells (RGCs) fail to regenerate following damage. As a result, RGCs die after acute injury and in progressive degenerative diseases such as glaucoma; this can lead to permanent vision loss and, eventually, blindness. Lipids are crucial for the development and maintenance of cell membranes, myelin sheaths, and cellular signaling pathways, however, little is known about their role in axon injury and repair. Studies examining changes to the lipidome during optic nerve (ON) regeneration could greatly inform treatment strategies, yet these are largely lacking. Experimental animal models of ON regeneration have facilitated the exploration of the molecular determinants that affect RGC axon regeneration. Here, we analyzed lipid profiles of the ON and retina in an ON crush rat model using liquid chromatography–mass spectrometry. Furthermore, we investigated lipidome changes after ON crush followed by intravitreal treatment with Zymosan, a yeast cell wall derivative known to enhance RGC regeneration. This data is available at the NIH Common Fund's Metabolomics Data Repository and Coordinating Center (supported by NIH grant, U01-DK097430) website, the Metabolomics Workbench, http://www.metabolomicsworkbench.org, where it has been assigned Project ID: PR000661. The data can be accessed directly via it's Project DOI: doi: 10.21,228/M87D53.

**Table:** Specifications table

Subject area	Biology
More specific subject area	Lipids
Type of data	Chromatograms, spectra, tables
How data was acquired	LC-MS/MS
Data format	Raw, filtered, analyzed
Experimental factors	Intact, optic nerve crush + PBS, optic nerve crush + Zymosan + CPT-cAMP
Experimental features	Rat optic nerves were collected 3, 7, 14 and retinas 7, 14 days post-crush. After a chloroform-methanol based extraction, lipid samples were analyzed using high performance liquid chromatography with C30 column coupled to a Q Exactive mass spectrometer operated in a data-dependent mode.
Data source location	Bascom Palmer Eye Institute, Miller School of Medicine at University of Miami, Miami, FL 33,136, USA and The Metabolomics Workbench
Data accessibility	The Metabolomics Workbench -PR000661, https://doi.org/10.21228/M87D53 Figshare -https://doi.org/10.6084/m9.figshare.7078253.v2
Related research article	Stark, D.T. et al., Optic Nerve Regeneration After Crush Remodels the Injury Site: Molecular Insights From Imaging Mass Spectrometry. Invest Ophthalmol Vis Sci, 2018.59(1): p. 212–222.

**Table:** 

**Value of the data** • The dataset can serve to inform future functional studies on the involvement of lipids in the RGCs injury and regeneration response. • The dataset provides the information of the expression of lipids present in the rat retina and ON at the baseline and over time during injury and repair. • Additionally, the data can be used to create lipid spectral libraries for the targeted lipidomic experiments.

## 1 Data

**Fig. 1:**
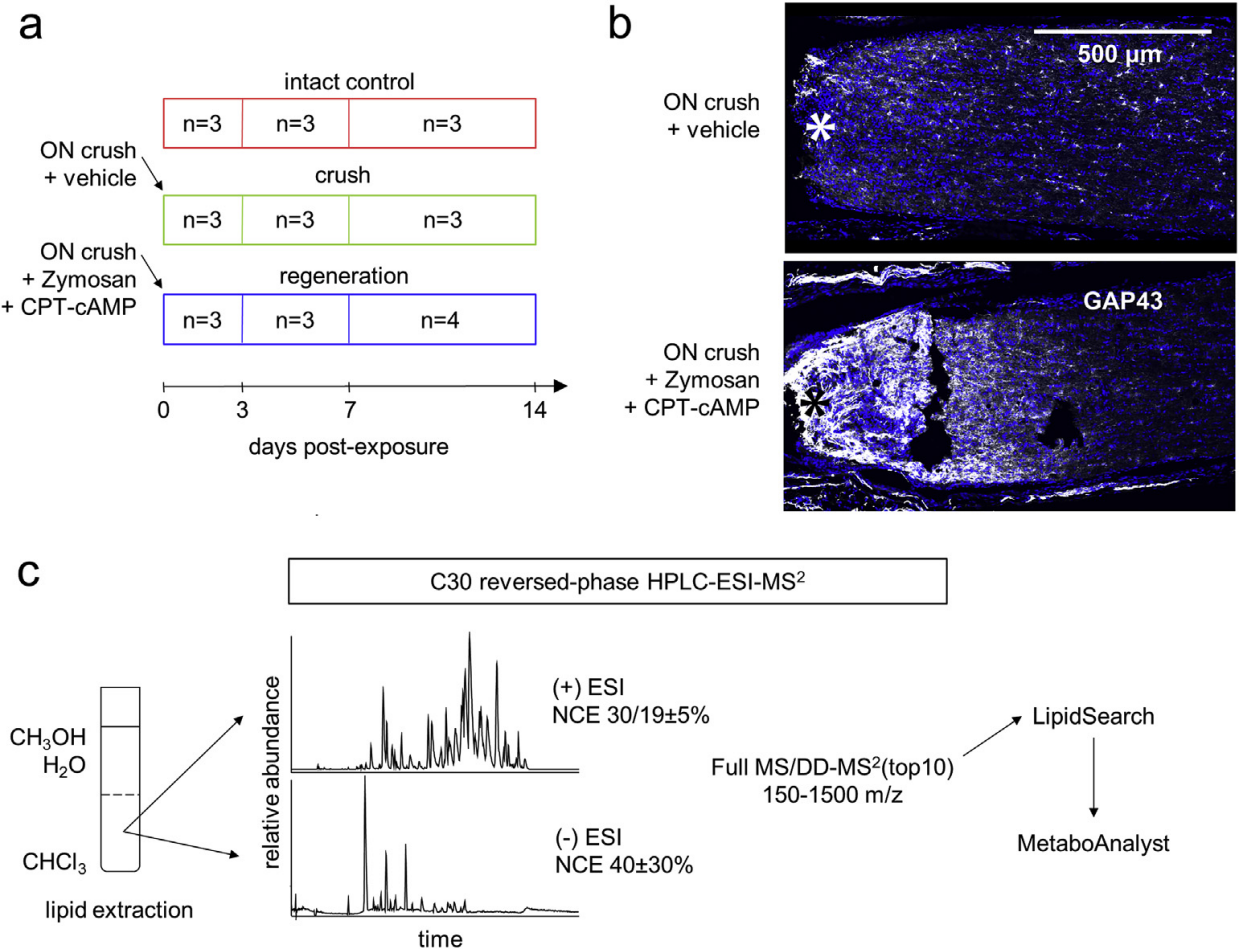
**Lipid profiling of the optic nerve (ON) regeneration**. (**a**) The dataset consists of the following experimental groups: intact control, ON crush (followed by intravitreal injection of the vehicle) and inflammation-induced ON regeneration (ON crush followed by intravitreal injection of Zymosan + CPT-cAMP). ONs were harvested 3, 7, 14 days post-crush and retinas 7, 14 days post-crush. (**b**) In the longitudinal sections of rat ON, Zymosan + CPT-cAMP increases expression of a marker of axon regeneration, GAP43, distal to the crush site (*). (**c**) Following methanol-chloroform-based extraction, lipids were separated by C30 high-performance liquid chromatography (HPLC) system using Accela 600 pump and measured in (+)/(−) heated electrospray (HESI) ionization mode using a Q Exactive mass spectrometer. Lipidome identification and relative quantification were performed in LipidSearch, followed by statistical analysis in MetaboAnalyst.

Lipid profiling was performed from the retina and ON samples during Zymosan-induced retinal ganglion cells regeneration through extractive mass spectrometry-based lipidomics. The experimental groups were: intact control (control), optic nerve (ON) crush + vehicle (crush) and ON crush + Zymosan + CPT-cAMP (regeneration). Zymosan is a yeast cell wall preparation traditionally used to induce sterile inflammation experimentally. The addition of a cell-permeable cAMP analog (CPT-cAMP) potentiates Zymosan's action but cannot induce ON regeneration when administered alone [1]. The ONs were collected 3, 7, 14 and the retinas 7, 14 days post-crush (Fig. 1a). Zymosan + CPT-cAMP treatment potently increased the amount of axon regeneration (Fig. 1b). Time points were chosen according to our and others previous reports. After axotomy, most RGCs die within 2 weeks [2]. The intravitreal inflammatory response presents a hazy vitreous on day 3 post-crush and concomitant Zymosan injection [3]. On day 7, the ON crush site is densely occupied by Iba1 positive macrophages/microglia [3]. Zymosan + CPT-cAMP doubles the number of live RGCs in retina 2 weeks after ON crush [1]. After a chloroform-methanol based extraction, lipid samples were analyzed using high-performance liquid chromatography (HPLC) with C30 column coupled to a Q Exactive mass spectrometer operated in a data-dependent mode (Fig. 1c). Peak identification and relative quantification were performed in LipidSearch software. Lists of species and their relative abundances were uploaded to MetaboAnalyst [4] for statistical analysis.

## 2 Experimental design, materials, and methods

### 2.1 ON crush and intravitreal injections

**Fig. 2:**
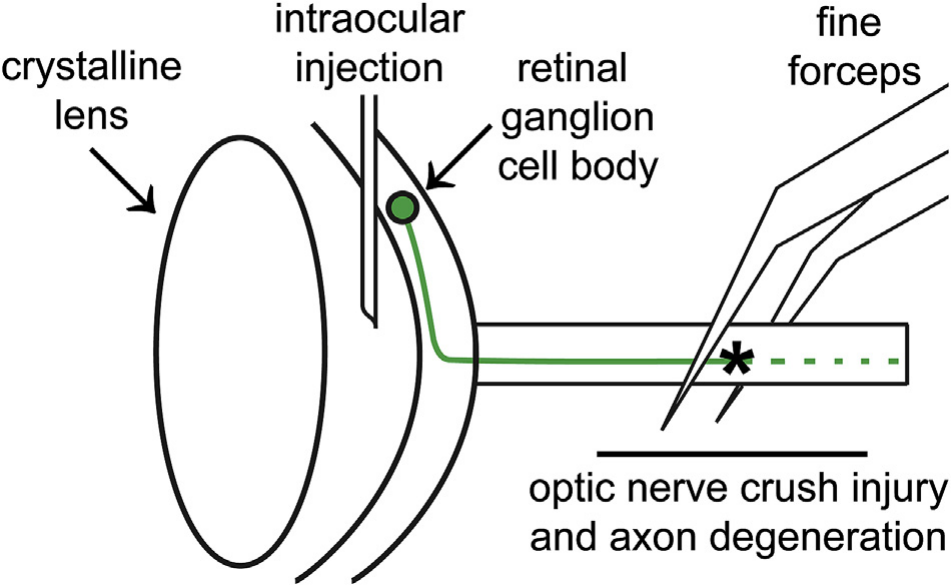
Schematic diagram of optic nerve crush and intravitreal injection.

All animal procedures were performed in accordance with the ARVO Statement for the Use of Animals in Ophthalmic and Vision Research and policies of the UCLA Animal Research Committee. A rat model of inflammation-induced ON regeneration was established with intravitreal injection of a yeast cell wall preparation (Zymosan A) [5] and a cell-permeant CPT-cAMP [1], immediately after ON crush. Ten-week-old male Fischer rats were deeply anesthetized by inhalation of isoflurane, and the eyes were treated with topical anesthetic (proparacaine HCl 0.5% ophthalmic) and a cycloplegic (tropicamide 0.5% ophthalmic) to reduce pain and assist with visualization of intravitreal injections. The left ON was exposed by blunt dissection through a temporal, fornix-based conjunctival incision and crushed for 10 seconds with Dumoxel #N5 self-closing forceps (Dumont, Montignez, Switzerland). Post crush, an absence of injury to the retinal vascular supply was confirmed by funduscopic examination. Intravitreal injections (5 μL) of PBS vehicle or a suspension of finely ground, sterilized 4 Zymosan A (Z4250; Sigma-Aldrich, St. Louis, MO, USA; 12.5 mg/mL) plus CPT-cAMP (C3912; Sigma-Aldrich, St. Louis, MO, USA; 100 μM) were performed with a pulled glass pipette attached to a Hamilton syringe on a manual micromanipulator. Injections were made 2 mm posterior to the limbus, and care was taken to prevent lens injury, choroidal hemorrhage, or retinal detachment. Post intravitreal injection, an absence of lens injury, choroidal haemorrhage and retinal detachment was confirmed by fundoscopic examination. Conjunctival incisions were closed with 8–0 polyglactin sutures and petrolatum ophthalmic ointment was applied to the ocular surface. Schematic diagram of optic nerve crush and intravitreal injection is presented in Fig. 2.

### 2.2 Immunohistochemistry

Optic nerves were fixed in PBS plus 4% paraformaldehyde and cryoprotected overnight at 4 °C in 30% sucrose. Cryoprotected tissue was embedded in optimal cutting temperature compound and flash frozen in liquid nitrogen. Longitudinal sections of nerve (14 μm) were cut with a cryostat, mounted on plus charged glass microscope slides, and permeabilized for 30 minutes at room temperature (RT) in TBS plus 0.25% Tween 20 (0.25%TBST). The sections were then blocked with 10% normal donkey serum in TBS for 1 hour at RT and incubated overnight at 4 °C with gentle shaking in 0.1%TBST plus 2% BSA (2%BSA-0.1%TBST) and rabbit polyclonal anti-GAP43 (Abcam, Cambridge, MA, USA; ab16053; 1:250). The sections were rinsed with 0.1%TBST and incubated for 1 hour at RT with Hoechst 33,258 in 2%BSA-0.1%TBST plus Alexa Fluor-conjugated donkey secondary antibody. Finally, the sections were rinsed with 0.1%TBST and coverslipped with an aqueous mounting medium. Images of immunostained tissues were obtained with a Fluoview FV1000 confocal microscope (Olympus, Center Valley, PA, USA).

### 2.3 Lipid extraction

Specimens were stored at −80 °C. 6 mL of methanol (LC-MS grade) and 3 mL of chloroform (LC-MS grade) were added to each sample. After 2 min of vigorous vortexing and 2 min of sonication in an ultrasonic bath, the samples were incubated at 48 °C overnight (in borosilicate glass vials, PTFE-lined caps). The following day, 3 mL of water (LC-MS grade) and 1.5 mL of chloroform were added, samples vigorously vortexed for 2 min and centrifuged at 3000 RCF, 4 °C for 15 min to obtain phase separation. Lower phases were collected and dried in a centrifugal vacuum concentrator. Samples were stored at −20 °C until reconstituted in 60 μL of chloroform:methanol (1:1) prior to mass spectrometric analysis.

### 2.4 High-performance liquid chromatography (HPLC)

Reversed-phase chromatographic separation was achieved with the Accela Autosampler, Accela 600 pump and Acclaim C30 column: 3 μm, 2.1 × 150 mm (Thermo Fisher Scientific, Waltham, MA). The column temperature was maintained at 30 °C (negative mode) or 45 °C (positive mode) and tray temperature at 20 °C. Solvent A was composed of 10 mM ammonium acetate (LC-MS grade) in 60:40 methanol:water (LC-MS grade) with 0.2% formic acid (LC-MS grade). Solvent B was composed of 10 mM ammonium acetate with 60:40 methanol:chloroform with 0.2% formic acid. The flow rate was 260 μL/min, and the injection volume was 5 μL. The gradient was 35–100% solvent B over 13.0 min, 100% solvent B over 13.0–13.8 min, 100-35% solvent B over 13.8–14.5 min, 35% solvent B over 14.5–18.0 min, 0% solvent B over 18.0–20.0 min.

### 2.5 Mass spectrometry

The Q Exactive (Thermo) mass spectrometer was operated under heated electrospray ionization (HESI) in positive and negative modes separately for each sample. The spray voltage was 4.4 kV, the heated capillary was held at 310 °C (negative mode) or 350 °C (positive mode) and heater at 275 °C (positive mode). The S-lens radio frequency (RF) level was 70. The sheath gas flow rate was 30 (negative mode) or 45 units (positive mode), and auxiliary gas was 14 (negative mode) or 15 units (positive mode). Full scan (m/z 150–1500) used resolution 70,000 at m/z 200 with automatic gain control (AGC) target of 1 × 10^6^ ions and maximum ion injection time (IT) of 100 ms. Data-dependent MS/MS (top 10) were acquired with the following parameters: resolution 17,500; AGC 2 × 10^5^ (negative mode) or 1 × 10^5^ (positive mode); maximum IT 100 ms (negative mode) or 75 ms (positive mode); 1.3 m/z isolation window. Normalized collision energy (NCE) settings were 40 ± 30% for the negative mode and 30, parallel with 19 ± 5% for the positive mode. Samples list is available in Table 1.

**Table 1:** Samples list.

Data	Subject	Eye	Tissue	Day	ON crush	IVI injection		Lipid extraction protocol	LC-MS protocol	ESI mode
						PBS	Zymosan +CPT-cAMP			
DS_1_POS	Rat_1	OD	ON	3	–	–	–	+	+	+
DS_1_NEG	Rat_1	OD	ON	3	–	–	–	+	+	–
DS_2_POS	Rat_2	OD	ON	3	–	–	–	+	+	+
DS_2_NEG	Rat_2	OD	ON	3	–	–	–	+	+	–
DS_3_POS	Rat_3	OD	ON	3	–	–	–	+	+	+
DS_3_NEG	Rat_3	OD	ON	3	–	–	–	+	+	–
DS_4_POS	Rat_1	OS	ON	3	+	+	–	+	+	+
DS_4_NEG	Rat_1	OS	ON	3	+	+	–	+	+	–
DS_5_POS	Rat_2	OS	ON	3	+	+	–	+	+	+
DS_5_NEG	Rat_2	OS	ON	3	+	+	–	+	+	–
DS_6_POS	Rat_3	OS	ON	3	+	+	–	+	+	+
DS_6_NEG	Rat_3	OS	ON	3	+	+	–	+	+	–
DS_7_POS	Rat_4	OS	ON	3	+	–	+	+	+	+
DS_7_NEG	Rat_4	OS	ON	3	+	–	+	+	+	–
DS_8_POS	Rat_5	OS	ON	3	+	–	+	+	+	+
DS_8_NEG	Rat_5	OS	ON	3	+	–	+	+	+	–
DS_9_POS	Rat_6	OS	ON	3	+	–	+	+	+	+
DS_9_NEG	Rat_6	OS	ON	3	+	–	+	+	+	–
DS_10_POS	Rat_7	OD	ON	7	–	–	–	+	+	+
DS_10_NEG	Rat_7	OD	ON	7	–	–	–	+	+	–
DS_11_POS	Rat_8	OD	ON	7	–	–	–	+	+	+
DS_11_NEG	Rat_8	OD	ON	7	–	–	–	+	+	–
DS_12_POS	Rat_9	OD	ON	7	–	–	–	+	+	+
DS_12_NEG	Rat_9	OD	ON	7	–	–	–	+	+	–
DS_13_POS	Rat_7	OS	ON	7	+	+	–	+	+	+
DS_13_NEG	Rat_7	OS	ON	7	+	+	–	+	+	–
DS_14_POS	Rat_8	OS	ON	7	+	+	–	+	+	+
DS_14_NEG	Rat_8	OS	ON	7	+	+	–	+	+	–
DS_15_POS	Rat_9	OS	ON	7	+	+	–	+	+	+
DS_15_NEG	Rat_9	OS	ON	7	+	+	–	+	+	–
DS_16_POS	Rat_10	OS	ON	7	+	–	+	+	+	+
DS_16_NEG	Rat_10	OS	ON	7	+	–	+	+	+	–
DS_17_POS	Rat_11	OS	ON	7	+	–	+	+	+	+
DS_17_NEG	Rat_11	OS	ON	7	+	–	+	+	+	–
DS_18_POS	Rat_12	OS	ON	7	+	–	+	+	+	+
DS_18_NEG	Rat_12	OS	ON	7	+	–	+	+	+	–
DS_19_POS	Rat_7	OD	retina	7	–	–	–	+	+	+
DS_19_NEG	Rat_7	OD	retina	7	–	–	–	+	+	–
DS_20_POS	Rat_8	OD	retina	7	–	–	–	+	+	+
DS_20_NEG	Rat_8	OD	retina	7	–	–	–	+	+	–
DS_21_POS	Rat_9	OD	retina	7	–	–	–	+	+	+
DS_21_NEG	Rat_9	OD	retina	7	–	–	–	+	+	–
DS_22_POS	Rat_7	OS	retina	7	+	+	–	+	+	+
DS_22_NEG	Rat_7	OS	retina	7	+	+	–	+	+	–
DS_23_POS	Rat_8	OS	retina	7	+	+	–	+	+	+
DS_23_NEG	Rat_8	OS	retina	7	+	+	–	+	+	–
DS_24_POS	Rat_9	OS	retina	7	+	+	–	+	+	+
DS_24_NEG	Rat_9	OS	retina	7	+	+	–	+	+	–
DS_25_POS	Rat_10	OS	retina	7	+	–	+	+	+	+
DS_25_NEG	Rat_10	OS	retina	7	+	–	+	+	+	–
DS_26_POS	Rat_11	OS	retina	7	+	–	+	+	+	+
DS_26_NEG	Rat_11	OS	retina	7	+	–	+	+	+	–
DS_27_POS	Rat_12	OS	retina	7	+	–	+	+	+	+
DS_27_NEG	Rat_12	OS	retina	7	+	–	+	+	+	–
DS_28_POS	Rat_13	OD	ON	14	–	–	–	+	+	+
DS_28_NEG	Rat_13	OD	ON	14	–	–	–	+	+	–
DS_29_POS	Rat_14	OD	ON	14	–	–	–	+	+	+
DS_29_NEG	Rat_14	OD	ON	14	–	–	–	+	+	–
DS_30_POS	Rat_15	OD	ON	14	–	–	–	+	+	+
DS_30_NEG	Rat_15	OD	ON	14	–	–	–	+	+	–
DS_31_POS	Rat_13	OS	ON	14	+	+	–	+	+	+
DS_31_NEG	Rat_13	OS	ON	14	+	+	–	+	+	–
DS_32_POS	Rat_14	OS	ON	14	+	+	–	+	+	+
DS_32_NEG	Rat_14	OS	ON	14	+	+	–	+	+	–
DS_33_POS	Rat_15	OS	ON	14	+	+	–	+	+	+
DS_33_NEG	Rat_15	OS	ON	14	+	+	–	+	+	–
DS_34_POS	Rat_16	OS	ON	14	+	–	+	+	+	+
DS_34_NEG	Rat_16	OS	ON	14	+	–	+	+	+	–
DS_35_POS	Rat_17	OS	ON	14	+	–	+	+	+	+
DS_35_NEG	Rat_17	OS	ON	14	+	–	+	+	+	–
DS_36_POS	Rat_18	OS	ON	14	+	–	+	+	+	+
DS_36_NEG	Rat_18	OS	ON	14	+	–	+	+	+	–
DS_37_POS	Rat_19	OS	ON	14	+	–	+	+	+	+
DS_37_NEG	Rat_19	OS	ON	14	+	–	+	+	+	–
DS_38_POS	Rat_13	OD	retina	14	–	–	–	+	+	+
DS_38_NEG	Rat_13	OD	retina	14	–	–	–	+	+	–
DS_39_POS	Rat_14	OD	retina	14	–	–	–	+	+	+
DS_39_NEG	Rat_14	OD	retina	14	–	–	–	+	+	–
DS_40_POS	Rat_15	OD	retina	14	–	–	–	+	+	+
DS_40_NEG	Rat_15	OD	retina	14	–	–	–	+	+	–
DS_41_POS	Rat_13	OS	retina	14	+	+	–	+	+	+
DS_41_NEG	Rat_13	OS	retina	14	+	+	–	+	+	–
DS_42_POS	Rat_14	OS	retina	14	+	+	–	+	+	+
DS_42_NEG	Rat_14	OS	retina	14	+	+	–	+	+	–
DS_43_POS	Rat_15	OS	retina	14	+	+	–	+	+	+
DS_43_NEG	Rat_15	OS	retina	14	+	+	–	+	+	–
DS_44_POS	Rat_16	OS	retina	14	+	–	+	+	+	+
DS_44_NEG	Rat_16	OS	retina	14	+	–	+	+	+	–
DS_45_POS	Rat_17	OS	retina	14	+	–	+	+	+	+
DS_45_NEG	Rat_17	OS	retina	14	+	–	+	+	+	–
DS_46_POS	Rat_18	OS	retina	14	+	–	+	+	+	+
DS_46_NEG	Rat_18	OS	retina	14	+	–	+	+	+	–
DS_47_POS	Rat_19	OS	retina	14	+	–	+	+	+	+
DS_47_NEG	Rat_19	OS	retina	14	+	–	+	+	+	–

### 2.6 Lipid identification and relative quantification

Lipid identification and relative quantification were performed with LipidSearch 4.1 software (Thermo). The search criteria were as follows: product search; parent m/z tolerance 5 ppm; product m/z tolerance 10 ppm; product ion intensity threshold 1%; filters: toprank, main isomer peak, FA priority; quantification: m/z tolerance 5 ppm, retention time tolerance 1 min. The following adducts were allowed in positive mode: +H, +NH4, +H—H2O, +H—2H2O, +2H, and negative mode: —H, +HCOO, +CH3COO, -2H. All classes were selected for search. LipidSearch nomenclature is used (Table 2).

**Table 2:** LipidSearch nomenclature of the identified lipid species.

Group	ClassKey	Lipid name
phospholipid	BisMePA	bismethyl phosphatidic acid
	cPA	cyclic phosphatidic acid
	dMePE	dimethylphosphatidylethanolamine
	LdMePE	lysodimethylphosphatidylethanolamine
	LPC	lysophosphatidylcholine
	LPE	lysophosphatidylethanolamine
	LPI	lysophosphatidylinositol
	PA	phosphatidic acid
	PC	phosphatidylcholine
	PE	phosphatidylethanolamine
	PEt	phosphatidylethanol
	PG	phosphatidylglycerol
	PI	phosphatidylinositol
	PMe	phosphatidylmethanol
	PS	phosphatidylserine
sphingolipid	Cer	ceramide
	SM	sphingomyelin
	So	sphingosine
glycosphingolipid	CerG1	hexosyl ceramide
	CerG3	trihexosyl ceramide
	SoG1	hexosyl sphingosine
	ST	sulfatide
cardiolipin	CL	cardiolipin
neutral glycerolipid	DG	diglyceride
	MG	monoglyceride
	TG	triglyceride
steroid	ChE	cholesterol ester
	ZyE	zymosterol
coenzyme	Co	coenzyme
fatty esters	AcCa	acyl carnitine
	WE	wax ester
glycoglycerolipid	MGDG	monogalactosyldiacylglycerol
	SQDG	sulfoquinovosyldiacylglycerol

### 2.7 Data processing

Positive and negative mode identifications of the retina and ON samples were aligned in LipidSearch 4.1, allowing calculation of unassigned peaks. The following settings were applied: product search; alignment method max; retention time tolerance 0.1 min; filters: toprank, main isomer peak; M-score 5. Only peaks with molecular identification grade: A-B (A: lipid class and fatty acid completely identified or B: lipid class and some fatty acid identified) were accepted. Only peaks appearing in all biological replicates were accepted. Peaks with the same annotated lipid species were merged. Lists of species and their relative abundances were uploaded to the MetaboAnalyst 4.0 [4] statistical analysis module.

### 2.8 Data availability

This data is available at the NIH Common Fund's Metabolomics Data Repository and Coordinating Center (supported by NIH grant, U01-DK097430) website, the Metabolomics Workbench, http://www.metabolomicsworkbench.org, where it has been assigned Project ID: PR000661. The data can be accessed directly via it's Project DOI: doi: 10.21,228/M87D53. The submission includes 94. RAW files: Q Exactive output (description of each file is in Table 1, Table 2. txt files: LipidSearch alignment output for retina and ON, 1. xlxs file: list of peaks (filtered) and 1. docx file: description of the method.

In addition, 10. csv files have been uploaded to Figshare https://doi.org/10.6084/m9.figshare.7078253.v2. Species input: ON3_MetaboAnalyst.csv, ON7_MetaboAnalyst.csv, ON14_MetaboAnalyst.csv, retina7_MetaboAnalyst.csv and retina14_MetaboAnalyst.csv. Classes input: ON3_classMetaboAnalyst.csv, ON7_classMetaboAnalyst.csv, ON14_classMetaboAnalyst.csv, retina7_classMetaboAnalyst.csv and retina14_classMetaboAnalyst.csv.

### 2.9 Usage notes

Files in. csv format can be directly input to MetaboAnalyst: Statistical Analysis. The user should select the following format: samples in columns (unpaired). For the following analysis example, we replaced missing values with a small number (half of the minimum positive value in the original data), applied normalization to sum and log 2 transformation.

**Fig. 3:**
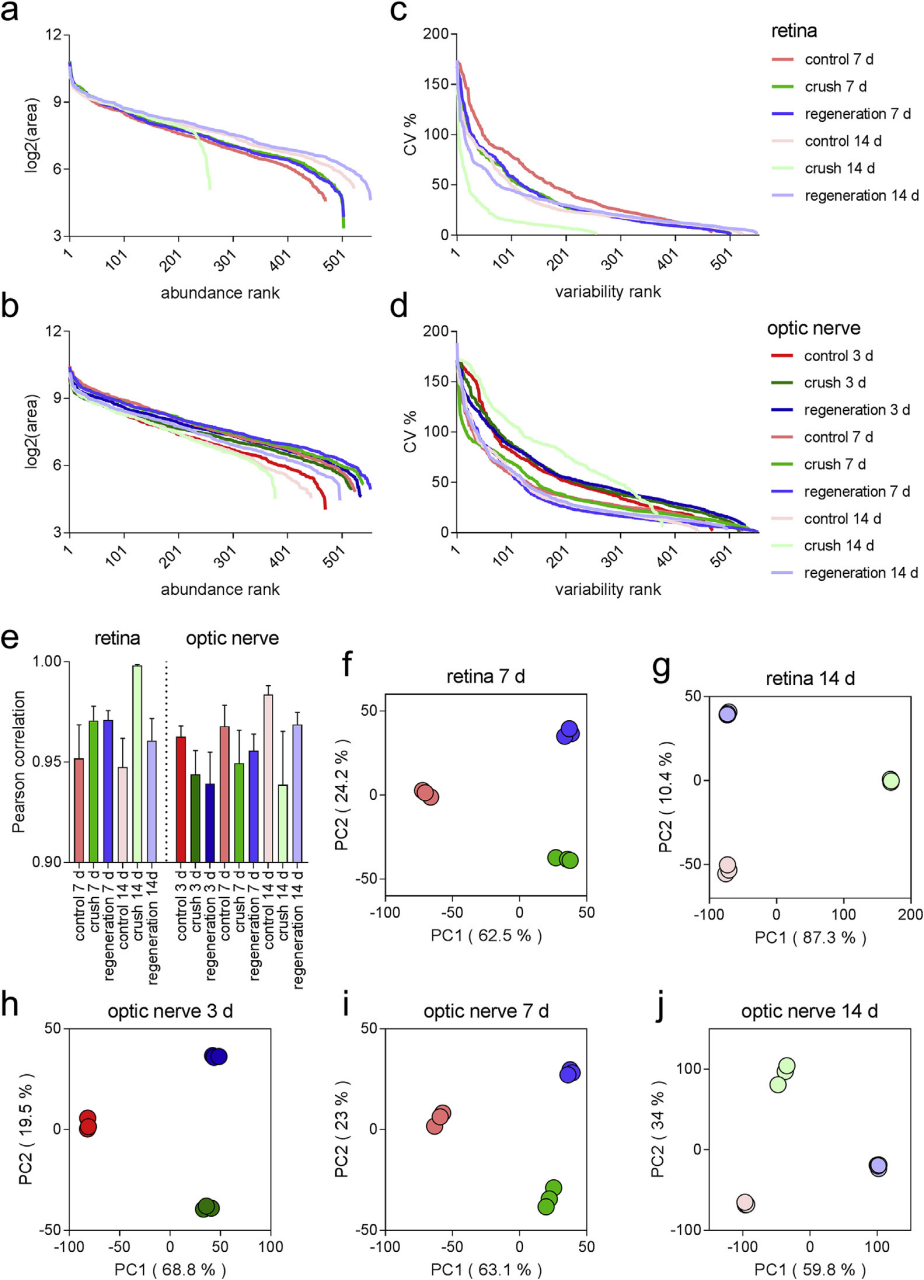
**Dataset overview**. (**a**–**b**) Abundance (average area) and (**c**–**d**) variability (% CV) distribution among treatment groups. Retina samples: (**a**–**c**). Optic nerve samples: (**b**–**d**). (**e**) Pearson's correlation coefficients between biological replicates within each treatment group. Mean + SD. (**f**–**j**) Principal component analysis (PCA) with samples plotted in 2 dimensions using their projection onto the first 2 principal components (in brackets % of total variance explained).

Data were obtained from 3 to 4 biological replicates for each group. In Fig. 3, distributions of the average area (Fig. 3a and b) and CV % values (Fig. 3c and d) for the experimental groups are presented. Biological replicates showed Pearson correlation coefficients ranging from 0.918 to 0.998 (Fig. 3e). In line, within each time point groups of samples were clearly distinguished from each other with 86.7–97.7% of variance accounted for by PC1 and PC2 (Fig. 3f and j).

**Fig. 4:**
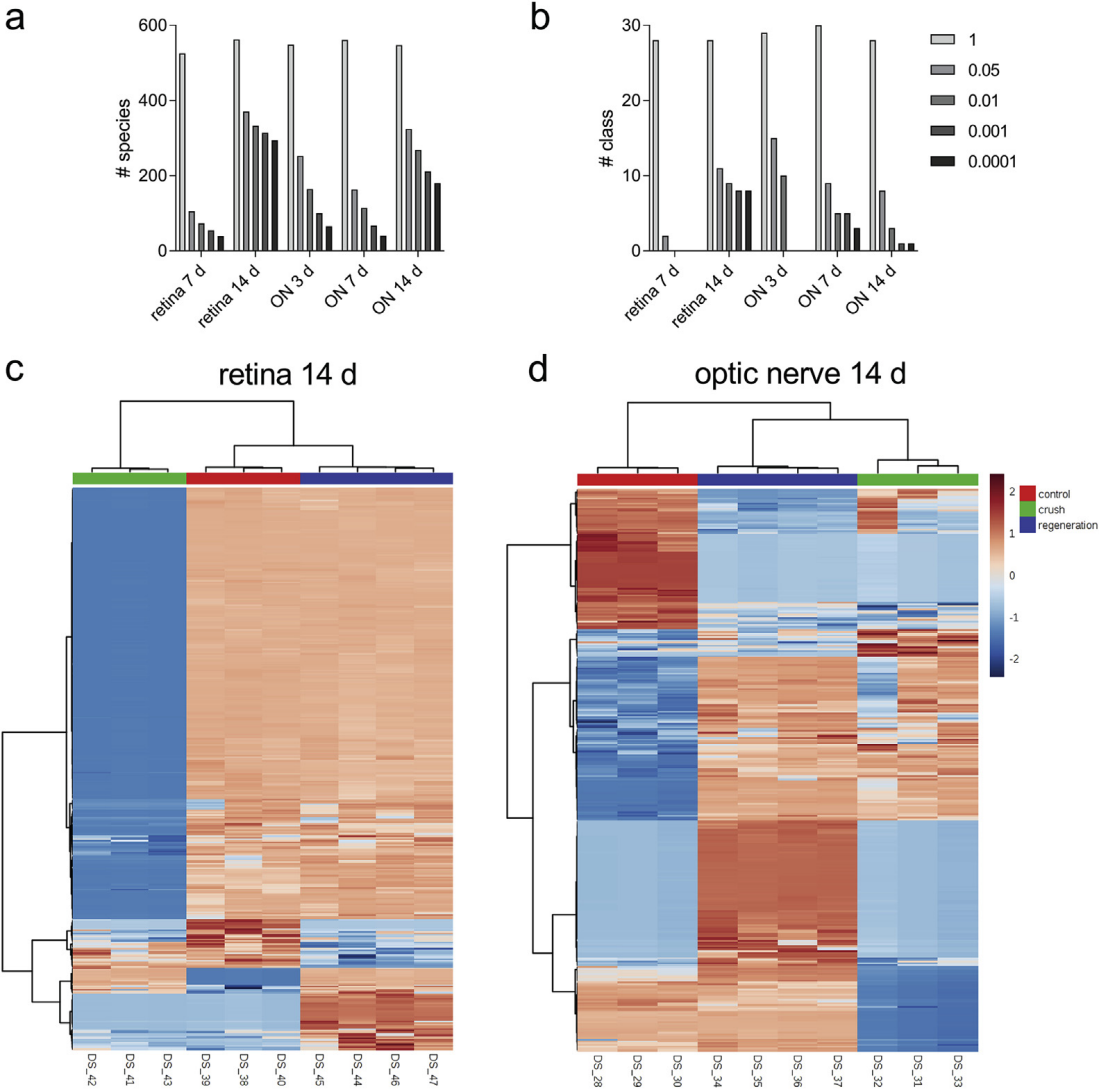
**Data analysis example**. (**a**–**b**) The number of significant features at different FDR adjusted p value cut-offs for one-way ANOVA analysis: species (**a**) and classes (**b**). (**c**–**d**) Accumulation change patterns of dysregulated species (FDR adjusted p-values <0.05) in the retina (370 species; **c**) and ON (324 species; **d**) 14 days post-crush, presented as heatmaps. Ward clustering algorithm, Euclidean distance measure, autoscale features. Species are not labeled for clarity.

To identify features undergoing the significant change between experimental groups, we used one-way ANOVA analysis with Tukey's post-hoc test. We examined a number of significant features at different FDR adjusted p values (Fig. 4 species **a** and classes **b**). Next, we performed hierarchical clustering and heatmap visualization of the dysregulated species (FDR adjusted p values < 0.05). The heatmaps of significant species in the retina and ON 14 days post-crush are presented as Fig. 4c and d, respectively.

## Author contributions

Conceptualization: D.S., S.K.B., J.C.

Methodology: D.S., M.P.

Investigation: A.T., D.S., J.K.

Analysis: A.T., D.S.

Writing – original draft: A.T., D.S.

Writing – review & editing: A.T., D.S., J.K., M.P., S.K.B., J.C.

Resources & Funding acquisition: S.K.B., J.C.

## References

[1] Kurimoto T. (2010). Long-distance axon regeneration in the mature optic nerve: contributions of Oncomodulin, cAMP, and pten gene deletion. J. Neurosci.: the official journal of the Society for Neuroscience.

[2] Kermer P. (2001). Transection of the optic nerve in rats: studying neuronal death and survival in vivo. Brain Res. Brain Res. Protoc..

[3] Stark D.T. (2018). Optic nerve regeneration after crush remodels the injury site: molecular insights from imaging mass spectrometry. Investig. Ophthalmol. Vis. Sci..

[4] Xia J., Wishart D.S. (2011). Web-based inference of biological patterns, functions and pathways from metabolomic data using MetaboAnalyst. Nat. Protoc..

[5] Yin Y. (2003). Macrophage-derived factors stimulate optic nerve regeneration. J. Neurosci..

