# Supplementary material for: Comparative lipid profiling dataset of the inflammation-induced optic nerve regeneration
Source: Data Brief. 2019 Apr 25;24:103950. doi: 10.1016/j.dib.2019.103950 (PMC6517575; doi:10.1016/j.dib.2019.103950)
Supplement: Multimedia component 1 [file mmc1.pdf]

Bascom Palmer Eye Institute
UNIVERSITY OF MIAMI HEALTH SYSTEM

To
The co-Editors-in-Chief/Managing Editor
Data-in-Brief

**Conflict of Interest Statement**

Manuscript No.: DIB-D-18-02776
Title: Comparative lipid profiling dataset of the inflammation-induced optic nerve regeneration Journal Title: Data in Brief Corresponding Author: Professor Sanjoy K. Bhattacharya All Authors: Anna Trzeciecka, MD; David T Stark, MD, PhD; Jacky M Kwong, PhD; Maria Piqueras, BS; Sanjoy K. Bhattacharya, Ph.D; Joseph Caprioli, MD
Submit Date: Oct 15, 2018

Dear Editor/Co-editors-in Chief,

On behalf of all authors, I declare that there is no conflict of interest whatsoever.

'Declarations of interest: none'.

Sincerely,

Sanjoy Bhattacharya, M. Tech., Ph. D.
Professor of Ophthalmology, Neuroscience, Biochemistry and Molecular Biology
Director, Miami Integrative Metabolomics Research Center (MIMRC)
